# Determination of yield losses against sheath rot caused by *Sarocladium oryzae* in rice varieties with differential resistance

**DOI:** 10.1038/s41598-025-05104-y

**Published:** 2025-10-17

**Authors:** Amritpal Mehta, S. K. Singh, Umer Basu, Shafat Ahmad Ahanger, Sonali Sharma, Bahaderjeet Singh, Amrish Vaid, Hayssam M. Ali, Waleed A. A. Alsakkaf, Ashwani Kumar Basandrai

**Affiliations:** 1 Division of Plant Pathology, Sher-e-Kashmir University of Agricultural Sciences and Technology of Jammu, Chatha, 180 009 India; 2 Department of Plant Pathology, Guru Kashi University, Talwandi Sabo, 151302 India; 3 Key Laboratory of Crop Stress Biology for Arid Areas, Key Laboratory of Plant Protection Resources and Pest Management of Ministry of Education, Key Laboratory of Integrated Pest Management on the Loess Plateau of Ministry of Agriculture and Rural Affairs, College of Plant Protection, Northwest A&F University, Yangling, 712100 Shaanxi China; 4 Division of Vegetable Science, Sher-e-Kashmir University of Agricultural Sciences and Technology of Jammu, Chatha, 180 009 India; 5 Former Principal Plant Pathologist cum Dean, College of Agriculture and College of Basic Sciences, CSK Himachal Pradesh Krishi Vishvavidyalaya, Palampur, 176062 Himachal Pradesh India; 6 Department of Botany and Microbiology, College of Science, King Saud University, Riyadh, 11451 Saudi Arabia

**Keywords:** Sheath rot, Resistance, Disease severity, Varieties, Integration, Crop loss model, Ecology, Plant sciences

## Abstract

Sheath rot (*Sarocladium oryzae*) of rice causes significant grain yield losses, warranting integrated, cost effective and eco-friendly management. With limited availability of highly resistant varieties, combining resistant / moderately resistant varieties with judicious and need based application of fungicides is a practical alternative. However, scarce information is available on the extent of yield losses in varieties with varied levels of resistance. This study evaluated yield losses in terms of 1000 grain weight (TGW) in rice varieties with varying resistance levels to sheath rot under protected conditions using fungicide azoxystrobin (11%) + tebuconazole (13.8%). It was observed that the differences in disease severity, AUDPC values and TGW loss were mainly attributed to the interaction effects among the treatment i.e. fungicide sprayed (protected) and fungicide non-sprayed (unprotected) treatment, and varieties & between varieties and years (*P* < 0.001). Plots with fungicidal application had a mean disease severity of 5.60% compared to 25.46% in untreated plots. Protected plots had a mean TGW of 24.80 g compared with 20.59 g in unprotected plots, reflecting a significant differences . Fungicide applications resulted in reduction of mean disease severity of 100, 74.03–97.57, 69.62–80.92, and 72.29–73.95% in resistant, moderately resistant, susceptible and highly susceptible varieties, respectively. A strong positive correlation was observed between disease severity and TGW loss during the cropping season 2019, 2020 and pooled data. The grain yield was enhanced, and crop loss models indicated good fitness with excellent predictive validity for estimating sheath rot impacts. As the first systematic assessment in northern region of India to systematically assess the impact of rice sheath rot. Its findings provide crucial insights into the interaction between fungicide treatments, varietal resistance, and disease dynamics, paving the way for more informed and effective management strategies in the region.

**Supplementary Information:**

The online version contains supplementary material available at 10.1038/s41598-025-05104-y.

## Introduction

Rice (*Oryza sativa* L.) is one of the most important staple food crops for over 1/3^rd^ of the world’s population. It is affected by numerous biotic and abiotic stresses, which lead to huge quantitative and qualitative losses. Among various biotic stresses, diseases viz. brown spot (*Bipolaris oryzae*; (Breda de Haan) Shoemaker), bacterial blight (*Xanthomonas oryzae* pv. *oryzae* (Ishiyama) Swings, van den Mooter, Vauterin, Hoste, Gillis, Mew & Kersters), blast (*Magnaporthe oryzae* (synonym of *Pyricularia oryzae *Sacc.)), false smut (*Ustilaginoidea virens* (Cooke) Takahashi), sheath blight (*Rhizoctonia solani* Kühn), sheath rot (*Sarocladium oryzae* (Sawada) Gams and Hawksworth) etc. pose a serious threat to its grain yield. Among these, sheath rot, previously considered as a minor disease, has emerged as a major threat in various rice growing regions globally^1–3^. The disease typically manifests during the booting or panicle initiation stage causing yield losses of 9.6–85%^4,5^.

Susceptibility to sheath rot is observed across diverse rice varieties, under both the rainfed and irrigated systems, with dwarf and high-yielding Asian varieties showing higher susceptibility^6^. Sheath rot symptoms appear on flag leaf sheath, a critical site for the translocation of non-structural carbohydrates to the panicle during grain filling. Specific infection at this site of the plant leads to hijacking of sugar by *S. oryzae*, causing accumulation of non-structural carbohydrates in the flag leaf sheath, which impairs grain filling and results in substantial yield losses^7^. Among various management practices, the use of resistant varieties is as the most economical and sustainable strategy, however, varieties with high level of resistance are not developed yet, and resistant donors are scarce^1,2^. In addition, most of commercially grown rice varieties in the production system of India are susceptible whereas, varieties with partial or moderate resistance have been reported^1,2,8^.

Fungicide application is an effective management strategy for managing sheath rot in rice, but the repeated use of fungicides could result in the development of pathogen resistance and adverse effects on human health, and the environment^9,10^. Consequently, the judicious use of fungicides along with agronomically desirable varieties with low/moderate level of resistance offers practical, cost effective and environmentally sustainable solution for managing this disease^1,11,12^. Research on this combinatorial rational strategy for sheath rot in rice is limited in the study areas and the country as well, and there is also lack of information on the correlation between disease severity and yield losses. Therefore, the present study was conducted to evaluate the effectiveness of combining host resistance with foliar fungicide applications for sheath rot management and quantify yield losses, measured as 1000-grain weight (TGW), under artificially inoculated conditions.

## Materials and methods

### Pathogen

The pathogen (*Sarocladium oryzae*) was isolated, purified and maintained following Ou^13^, Gams and Hawksworth^14^, and Mew and Mishra^15^. Among 31 isolates collected from various rice growing regions of Jammu-India, the isolate ‘SO_1_’ was selected for the present study due to its high virulence and wide prevalence in the region Mehta et al.^16^. The isolate was mass multiplied on rice grain media. The rice grain media were prepared as follows: thirty gram of rice grains were transferred into 250 ml conical flask and were soaked for 24 h in tap water to allow hydration. The excess water was drained off, and grains were double sterilized by autoclaving at 121 °C at 15 psi for 30 min. The sterilized grains were inoculated aseptically with the mycelial bit of *S. oryzae* grown on PDA medium and incubated at 28 ± 2 °C for 10 days, and the flasks were shaken every alternate day to avoid clumping. After 10 days, the rice grains were completely covered with whitish fungal mycelium and were used for inoculation by placing the infected rice grains singly in between the flag leaf sheath and un-emerged panicles at pre-emergence stage for the disease development^1^.

### Host (Rice varieties)

Forty-four varieties, including commercially grown basmati and non-basmati varieties with different resistance status i.e. resistant (5), moderately resistant (16), susceptible (20) and highly susceptible (3) were used in the study. The pedigree, origin and disease reaction of the varieties are provided in supplementary table 1.

### Field experiments

Field trials were conducted during *kharif* (i.e. June-July to October–November) cropping seasons of 2019 and 2020 at the Experiential Fields of Department of Plant Pathology, SKUAST-Jammu at Chatha on the soil type clay to clay loam. The experimental field was located at a latitude of 32° 40’ N, longitude of 74° 58’ E, with an elevation of 332 m above mean sea level. The experiments were laid out in a randomized block design (RBD) with three replications. Each replication comprised 44 varieties, transplanted in six rows of 2 m length (3 rows for sprayed plants and 3 for non-sprayed plants) with spacing of 20 cm between rows and 15 cm between plants. The application rates of nitrogen (120 kg/ha), phosphorus (60 kg/ha) and potash (30 kg/ha) were based on the recommendations of SKUAST-Jammu. Phosphorus, potash and 1/3^rd^ of the nitrogen dose was applied before transplanting, while the remaining nitrogen was administered in two equal splits at two and four weeks after transplanting. For the control of stem borer and leaf folder, cartap hydrochloride was applied at the rate of 25 kg/ha whereas, for the weed control two hand weedings (1st weeding was done 15 days after transplanting of the crop and 2nd weeding was done after a fortnight of first weeding) were given at 15 days intervals (Microsoft Word—POP Kharif_11.05.2016).

At the panicle initiation stage, twenty-five plants of each cultivar in both protected and unprotected plots were randomly selected, tagged and inoculated as described by Mehta et al.^1^. The protected plots were sprayed twice with azoxystrobin (11%) + tebuconazole (13.8%) SC at a rate of 0.1% at panicle initiation stage and 15 day later. Fungicide was applied during calm evening hours and a 2 m tall plastic sheet was placed around the sprayed plots to localize application. The nozzle was held close to the canopy to minimize spray drift and ensure precise targeting of the plants. The sheeting was left in place for about 30 min post-application to allow fungicide to settle on the canopy of the targeted plot.

Disease reaction data were recorded on a 0–9 scale^17^ at weekly intervals after 5 days of inoculation with four observations taken for both unprotected and protected plots until the crop maturation. Where, ‘0’ = No lesions on flag leaf sheath (Immune/free); ‘1’ =  < 1% of the flag leaf sheath area affected (Highly resistant); ‘3’ = 1%–5% area affected (Resistant); ‘5’ = 6%–25% area affected (Moderately resistant); ‘7’ = 26%–50% area affected (Susceptible) and ‘9’ = 51%– 100% area affected (Highly susceptible). Disease reaction data was used to calculate disease severity using the formula given by Mckinney^18^.

$${\text{Disease}}\;{\text{severity}} = \frac{{{\text{Sum}}\;{\text{of}}\;{\text{all}}\;{\text{numerical}}\;{\text{values}}\;({\text{disease}}\;{\text{reaction}})}}{{{\text{Number}}\;{\text{of}}\;{\text{diseased}}\;{\text{plants}}\;{\text{observed}}\;({\text{at}}\;{\text{one}}\;{\text{assessment}}\;{\text{time}}) \times {\text{maximum}}\;{\text{score}}}} \times 100$$

The Area under Disease Progress Curve (AUDPC) was calculated using the formula by Shaner and Finney^19^.

$${\text{AUDPC}}={\sum}_{i=1}^{n-1}({x}_{i}+{x}_{i+1})/2 \times ({t}_{i+1}-{t}_{i})$$

where *x*_*i*_ = disease severity (%) at the *i*th observation,

*t*_*i*_ = is time (days) at the *i*th observation,

*i* = 1….n—number of observations.

Infection rate ‘r’ was calculated using the formula of Vander Plank^20^.

$${\text{Infection}}\;{\text{rate}}\left( r \right) = \frac{{2.3}}{{t_{2} - t_{1} }} \times \log ^{{10}} \frac{{x_{2} (1 - x_{1} )}}{{x_{1} (1 - x_{2} )}}$$

where, *x*_*1*_ = area of infected tissues at time *t*_*1*_*.*

*x*_*2*_ = area of infected tissues at time *t*_*2*_*.*

*t*_*2*_*-t*_*1*_ = time interval between initial disease severity and final disease severity in days.

At maturity, 10 tagged plants from both unprotected and protected plots of each cultivar were harvested. The grain yield component of the rice varieties was estimated based on 1000 grain weight (TGW). The TGW loss was calculated using formula^21^:

$$1000{\text{ grain weight loss }}\left( {{\text{TGW}}} \right){ } = { }\frac{{{\text{Grain weight of healthy plants }}{-}{\text{ grain weight of diseases plants}}}}{{\text{Grain weight of healthy plants}}} \times 100$$

### Correlation and regression analysis

The correlation coefficient (r) of disease severity with TGW loss was calculated according to Pearson and Hartley^22^. A linear regression (LR) model was used to predict TGW loss as a function of disease severity:

$${\text{Y}} = b_{0} + b_{1} X_{1}$$

where, Y = TGW loss (predicted), ‘*b*_*0*_’ = intercept, *b*_*1*_ is the partial regression coefficient, and X_1_ = disease severity.

To determine the forecast errors of TGW loss during 2019 and 2020 cropping seasons, the differences between the actual and forecasted values from the LR model were used. Accuracy of the forecast model was ascertained by computing the values of the forecasting properties of the models^23^. Mean Absolute Percent Error (MAPE) was calculated to determine forecast errors. MAPE can be interpreted as the average percentage difference between the predicted and the actual values^23^. Peng et al.^24^ established values for a valid MAPE evaluation where, error of < 10, 10–20, and 20–30% infers that the model is perfect fit, good fit, acceptance accuracy, respectively and the error of > 30% indicated poor accuracy of the model.

$$MAPE = \frac{1}{N}\mathop \sum \limits_{i = 1}^{n} \frac{{\left( {Oi{ }{-}{ }Pi} \right)}}{Oi}$$

*O*_*i*_ = actual value,* P*_*i*_ = forecasted value, N = number of observations.

### Statistical analysis

The effect of different varieties, treatments (protected and unprotected) and the cropping seasons (2019 and 2020) on disease severity of sheath rot and TGW loss was determined by analysis of variance (ANOVA). ANOVA was performed using OPSTAT software (https://opstat.somee.com/opstat/) to determine significant differences among main factors (varieties, treatments and years) and their interactions. Disease severity and TGW loss were treated as dependent variables with varieties, treatments and years as independent variables. Correlation of disease severity with TGW loss, as well as the assumptions of LR analysis were calculated by using R studio software^25^. Predictive values of TGW loss were computed using ggcoefstats (selectmod) function in R-studio. Observed and predicted values of disease severity were plotted in Excel to evaluate the accuracy of model in predicting the TGW loss during 2019 and 2020 cropping seasons, as well as mean over the seasons.

## Results

### Disease severity

**Fig. 1:**
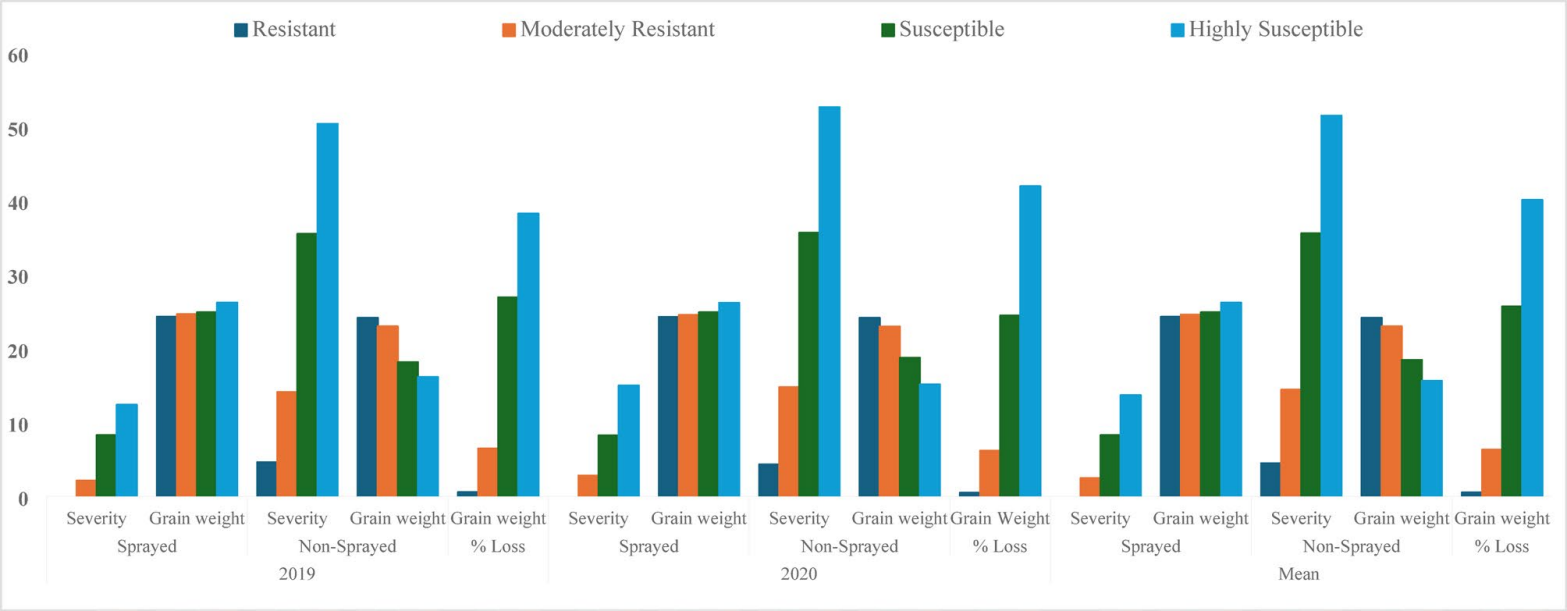
Mean disease severity of sheath rot, thousand grain weight (TGW) in sprayed (protected) and non-sprayed (unprotected) plot and, TGW loss during 2019, 2020 and mean over the years.

**Fig. 2:**
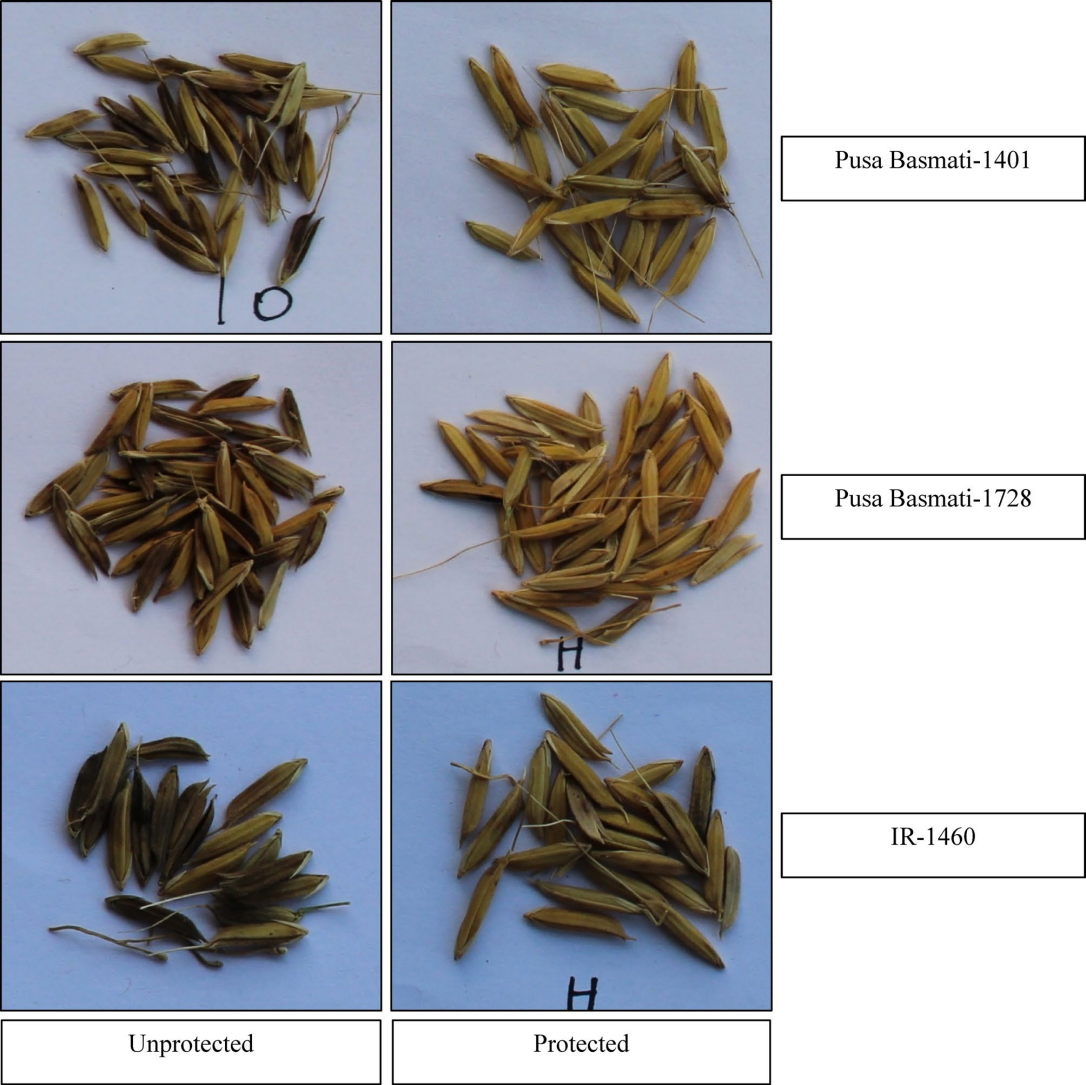
Pictorial representation of rice grains as affected by sheath rot under unprotected and fungicide protected treatment in different.

Among the 44 rice varieties evaluated for sheath rot disease reaction, 5, 16, 20, and 3 were resistant, moderately resistant, susceptible and highly susceptible, respectively (Table 1; Figs. 1, 2). During the cropping season 2019 and 2020 terminal disease severity (TDS) varied significantly across varieties (weather data; Supplementary Table 2 and 3). The data on disease severity was recorded on weekly intervals with TDS recorded thirty days after inoculation. The TDS was significantly lower in protected plots, but the differences in TDS among treatments, varieties and their interactions were highly significant (*P* < 0.001) during both cropping seasons.

**Table 1:** Terminal disease severity of sheath rot (*S. oryzae*) in different rice varieties during *Kharif* 2019 and 2020.

S. No	Varieties	Disease severity (%)								
		2019			2020			Mean		
		Protected	Un-Protected	% Reduction	Protected	Un-Protected	% Reduction	Protected	Un-Protected	% Reduction
1	K-448 (R)	0.00	5.71	100.00	0.00	4.12	100.00	0.00	4.92	100.00
2	Basmati-123 (R)	0.00	3.52	100.00	0.00	4.43	100.00	0.00	3.98	100.00
3	K-39 (R)	0.00	4.52	100.00	0.00	3.73	100.00	0.00	4.13	100.00
4	SJR-5 (R)	0.00	5.12	100.00	0.00	4.19	100.00	0.00	4.66	100.00
5	Pusa-44 (R)	0.00	4.33	100.00	0.00	5.22	100.00	0.00	4.78	100.00
	Mean	0.00	4.64	100.00	0.00	4.34	100.00	0.00	4.49	100.00
6	Sanwal-Basmati (MR)	2.95	16.15	81.73	2.45	13.57	81.95	2.70	14.86	81.83
7	Basmati-386 (MR)	1.05	15.63	93.28	1.65	16.75	90.15	1.35	16.19	91.66
8	K-343 (MR)	2.52	11.42	77.93	0.57	8.12	92.98	1.55	9.77	84.14
9	PR-118 (MR)	4.29	24.44	82.45	5.21	23.83	78.14	4.75	24.14	80.32
10	CR-212 (MR)	0.88	10.50	91.62	1.11	10.70	89.63	1.00	10.60	90.57
11	PR-126 (MR)	0.00	6.38	100.00	0.34	7.61	95.53	0.17	7.00	97.57
12	Fateh (MR)	1.05	8.33	87.39	1.22	11.11	89.02	1.14	9.72	88.27
13	PR-121 (MR)	1.21	11.82	89.76	1.54	12.68	87.85	1.38	12.25	88.73
14	SJR-51 (MR)	4.95	21.30	76.76	5.65	20.00	71.75	5.30	20.65	74.33
15	Peeli Pusa (MR)	3.28	18.36	82.14	6.23	20.44	69.52	4.76	19.40	75.46
16	CR-212 (MR)	0.62	9.40	93.40	0.45	10.10	95.54	0.54	9.75	94.46
17	Jaya (MR)	0.95	11.10	91.44	1.03	10.40	90.10	0.99	10.75	90.79
18	Punjab Basmati-1 (MR)	5.00	20.32	75.39	6.21	22.87	72.85	5.61	21.60	74.03
19	Giza-14 (MR)	0.50	10.70	95.33	0.87	11.30	92.30	0.69	11.00	93.73
20	Pusa-Sugandha (MR)	2.00	15.32	86.95	4.23	16.98	75.09	3.12	16.15	80.68
21	CSR-30 (MR)	3.02	15.02	79.89	6.12	20.33	69.90	4.57	17.68	74.15
	Mean	2.14	14.14	86.59	2.81	14.80	83.89	2.48	14.47	85.05
22	Pusa Basmati-1612 (S)	6.23	31.42	80.17	7.45	32.30	76.93	6.84	31.86	78.53
23	Ranbir-Basmati (S)	9.55	45.34	78.94	10.43	46.33	77.49	9.99	45.84	78.21
24	Pusa Basmati-1509 (S)	10.43	38.34	72.80	8.43	37.57	77.56	9.43	37.96	75.16
25	Basmati-370 (S)	8.03	34.38	76.64	6.43	31.11	79.33	7.23	32.75	77.92
26	Basmati-129 (S)	11.22	43.50	74.21	9.89	45.33	78.18	10.56	44.42	76.23
27	Arize 6444 Gold (S)	4.30	23.56	81.75	5.34	26.96	80.19	4.82	25.26	80.92
28	PR-114 (S)	5.55	26.40	78.98	6.23	27.44	77.30	5.89	26.92	78.12
29	PR-124 (S)	8.52	29.17	70.79	7.12	31.11	77.11	7.82	30.14	74.05
30	Kohinoor (S)	9.64	37.33	74.18	8.34	38.44	78.30	8.99	37.89	76.27
31	PR-127 (S)	8.52	32.40	73.70	6.12	29.70	79.39	7.32	31.05	76.43
32	432 (S)	5.05	27.45	81.60	5.32	26.19	79.69	5.19	26.82	80.65
33	PR-128 (S)	8.22	37.85	78.28	6.54	30.13	78.29	7.38	33.99	78.29
34	Basmati-564 (S)	8.43	31.42	73.17	7.12	32.37	78.00	7.78	31.90	75.61
35	PC-19 (S)	6.22	28.09	77.86	6.87	30.14	77.21	6.55	29.12	77.51
36	PR-129 (S)	13.21	47.40	72.13	16.21	49.43	67.21	14.71	48.42	69.62
37	PR-111 (S)	5.87	29.53	80.12	5.87	28.25	79.22	5.87	28.89	79.68
38	PR-113 (S)	10.25	43.65	76.52	11.65	45.56	74.43	10.95	44.61	75.45
39	Ratna (S)	8.21	35.87	77.11	7.13	33.42	78.67	7.67	34.65	77.86
40	Pusa Basmati-1728 (S)	9.23	41.45	77.73	10.43	44.33	76.47	9.83	42.89	77.08
41	IR-1460 (S)	9.24	45.40	79.65	12.54	47.23	73.45	10.89	46.32	76.49
	Mean	8.30	35.50	76.82	8.27	35.67	77.22	8.29	35.59	77.00
42	PR-122 (HS)	13.00	49.33	73.65	15.32	52.71	70.94	14.16	51.02	72.25
43	Pusa Basmati-1401 (HS)	12.22	49.58	75.35	14.54	53.14	72.64	13.38	51.36	73.95
44	Pusa Basmati-1121 (HS)	12.00	52.28	77.05	15.23	52.10	70.77	13.62	52.19	73.90
	Mean	12.41	50.40	75.35	15.03	52.65	71.45	13.72	51.52	73.37
	Overall Mean	5.40	25.24	78.61	5.81	25.68	77.38	5.60	25.46	78.00
Source of Variation		*LSD*	*SE*	Significance	*LSD*	*SE*	Significance	*LSD*	*SE*	Significance
Treatment		0.49	0.17	***	0.60	0.21	***	0.38	0.14	***
Varieties		2.28	0.82	***	2.81	1.01	***	1.80	0.64	***
Year								0.38	0.14	*
Treatment X Varieties		3.23	1.16	***	3.97	1.42	***	2.54	0.91	***
Treatment X Year								NA	0.19	NS
Varieties X Year								2.54	0.91	**
Treatment X Varieties X Year								NA	1.29	NS

*LSD* least significant difference at 0.05%, Significance = *P* < 0.05 (*), *P* < 0.01 (**), *P* < 0.001 (***), *SE* standard error, *R* resistant, *MR* moderately resistant, *S* susceptible, *HS* highly susceptible.

During 2019 and 2020 cropping seasons, unprotected plots recorded higher mean TDS values of 25.24 and 25.68%, respectively, whereas protected plots recorded mean TDS values of 5.40 and 5.81%, respectively resulting 78.61 and 77.38% reductions, respectively. TDS values in unprotected plots ranged from 3.52–52.28% to 3.73–53.14% during 2019 and 2020 cropping seasons, respectively. In 2019 the highest TDS was recorded in Pusa Basmati-1121 (52.28%), followed by Pusa Basmati-1401 (49.58%), PR-122 (49.33%), PR-129 (47.40%), IR-1460 (45.40%), Ranbir-Basmati (45.34%), PR-113 (43.65%), Basmati-129 (43.50%), and Pusa Basmati-1728 (41.45%). During the 2020 cropping season, variety Pusa Basmati-1401 (53.14%) developed the highest TDS followed by PR 122 (52.71%), Pusa Basmati-1121 (52.10%), PR-129 (49.43%), IR-1460 (47.23%), Ranbir-Basmati (46.33%), PR-113 (45.56%), Basmati-129 (45.33%), and Pusa Basmati-1728 (44.33%). The minimum TDS during cropping season 2019 was recorded in Basmati-123 (3.52%), K-39 (4.52%), Pusa-44 (4.33%), SJR-5 (5.12%), and K-448 (5.71%) while in 2020, it was minimum in varieties K-39 (3.73%) followed by SJR 5 (4.19%), K-448 (4.12%), Basmati-123 (4.43%), and Pusa-44 (5.22%) (Table 1; Fig. 1).

The observed differences in TDS were mainly attributed to the interaction effects between treatments and varieties as well as between varieties and cropping seasons (years) (*P* < 0.001). Protected plots showed mean TDS of 5.60% compared with 25.46% in unprotected plots with a reduction of 78.00%. TDS in highly susceptible, susceptible, moderately resistant and resistant varieties was reduced by 72.25–73.95%, 69.62–80.92%, 74.03–97.57%, and 100%, respectively.

### Area under disease progress curve (AUDPC)

The AUDPC values between protected and unprotected treatments, as well as their interaction effects were highly significant (*P* < 0.001) during both years.

In 2019 cropping season, unprotected plots had higher mean AUDPC values (316.75) compared to protected plots (52.40), which showed a significant reduction of 86.97% in AUDPC due to fungicide application. AUDPC in the unprotected varieties ranged between 39.97 and 688.46, with the highest AUDPC in variety Pusa Basmati-1121 (688.46) followed by PR-122 (656.24), Pusa Basmati-1401 (643.48), Basmati-129 (568.30), PR-129 (564.73), IR-1460 (559.07), and Pusa Basmati-1728 (499.16). The lowest AUDPC values were recorded in Pusa-44 (39.97) followed by K-448 (48.77), SJR-5 (49.47), K-39 (53.77), and Basmati-123 (57.25) (Table 2).

**Table 2:** Data on area under disease progress curve (AUDPC) of sheath rot (*S. oryzae*) in different rice varieties during *Kharif* 2019 and 2020.

S. No	Varieties		AUDPC Values							
		2019			2020			Mean over the years		
		Protected	Un-Protected	% Reduction	Protected	Un-Protected	% Reduction	Protected	Un-Protected	% Reduction
1	K-448 (R)	0.00	48.77	100.00	0.00	57.67	100.00	0.00	53.22	100.00
2	Basmati-123 (R)	0.00	57.25	100.00	0.00	46.83	100.00	0.00	52.04	100.00
3	K-39 (R)	0.00	53.77	100.00	0.00	58.82	100.00	0.00	56.3	100.00
4	SJR-5 (R)	0.00	49.47	100.00	0.00	57.13	100.00	0.00	53.3	100.00
5	Pusa-44 (R)	0.00	39.97	100.00	0.00	47.34	100.00	0.00	43.66	100.00
	Mean	0.00	49.85	100.00	0.00	53.56	100.00	0.00	51.70	100.00
6	Sanwal-Basmati (MR)	27.83	251.03	88.92	19.08	234.16	91.85	23.45	242.60	90.33
7	Basmati-386 (MR)	14.18	154.96	90.85	16.28	161.91	89.95	15.23	158.43	90.39
8	K-343 (MR)	26.32	188.08	86.01	5.85	158.45	96.31	16.08	173.26	90.72
9	PR-118 (MR)	25.52	279.14	90.86	28.74	238.86	87.97	27.13	259.00	89.53
10	CR-212 (MR)	10.08	150.87	93.32	10.89	122.41	91.11	10.48	136.64	92.33
11	PR-126 (MR)	0.00	111.35	100.00	1.19	112.15	98.94	0.60	111.75	99.47
12	Fateh (MR)	14.18	178.63	92.06	14.77	186.46	92.08	14.47	182.54	92.07
13	PR-121 (MR)	14.74	205.92	92.84	15.89	186.53	91.48	15.31	196.22	92.20
14	SJR-51 (MR)	45.33	304.00	85.09	65.28	293.78	77.78	55.30	298.89	81.50
15	Peeli Pusa (MR)	21.98	210.89	89.58	32.31	240.42	86.56	27.14	225.65	87.97
16	CR-212 (MR)	5.32	153.85	96.54	4.38	169.53	97.42	4.85	161.69	97.00
17	Jaya (MR)	9.28	120.04	92.27	12.36	108.59	88.62	10.82	114.32	90.54
18	Punjab Basmati-1 (MR)	56.00	247.29	77.35	67.24	264.12	74.54	61.62	255.70	75.90
19	Giza-14 (MR)	4.55	123.78	96.32	9.35	132.66	92.96	6.95	128.22	94.58
20	Pusa-Sugandha (MR)	7.00	260.07	97.31	25.31	290.38	91.29	16.15	275.23	94.13
21	CSR-30 (MR)	21.07	274.75	92.33	31.92	307.63	89.62	26.50	291.19	90.90
	Mean	18.96	200.92	91.35	22.55	200.50	89.91	20.76	200.71	90.60
22	Pusa Basmati-1612 (S)	85.93	491.23	82.51	89.01	532.02	83.27	87.47	511.62	82.90
23	Ranbir-Basmati (S)	99.51	442.42	77.51	92.51	433.31	78.65	96.01	437.87	78.07
24	Pusa Basmati-1509 (S)	67.31	368.54	81.74	71.58	400.79	82.14	69.44	384.66	81.95
25	Basmati-370 (S)	84.11	416.35	79.80	57.51	382.27	84.96	70.81	399.31	82.27
26	Basmati-129 (S)	112.77	568.30	80.16	101.12	595.70	83.03	106.94	582.00	81.63
27	Arize 6444 Gold (S)	25.55	252.82	89.89	29.19	288.52	89.88	27.37	270.67	89.89
28	PR-114 (S)	29.93	390.89	92.34	32.31	422.53	92.35	31.12	406.71	92.35
29	PR-124 (S)	85.82	344.87	75.12	79.17	365.66	78.35	82.50	355.26	76.78
30	Kohinoor (S)	93.24	403.74	76.91	88.69	439.27	79.81	90.97	421.50	78.42
31	PR-127 (S)	85.82	387.67	77.86	66.92	420.25	84.08	76.37	403.96	81.09
32	432 (S)	28.18	406.23	93.06	29.12	375.28	92.24	28.65	390.76	92.67
33	PR-128 (S)	39.27	438.82	91.05	33.39	375.98	91.12	36.33	407.40	91.08
34	Basmati-564 (S)	93.56	433.67	78.43	85.12	457.93	81.41	89.34	445.80	79.96
35	PC-19 (S)	77.77	321.16	75.78	88.45	354.31	75.04	83.11	337.74	75.39
36	PR-129 (S)	151.24	564.73	73.22	182.74	586.18	68.83	166.99	575.45	70.98
37	PR-111 (S)	69.55	325.51	78.64	62.55	338.45	81.52	66.05	331.98	80.11
38	PR-113 (S)	107.98	462.25	76.64	107.28	488.59	78.04	107.63	475.42	77.36
39	Ratna (S)	77.74	407.72	80.93	91.46	383.10	76.13	84.60	395.41	78.61
40	Pusa Basmati-1728 (S)	98.81	499.16	80.21	117.01	522.98	77.63	107.91	511.07	78.89
41	IR-1460 (S)	105.84	559.07	81.07	138.39	585.20	76.35	122.12	572.13	78.66
	Mean	81.00	424.26	81.14	82.18	437.42	81.74	81.59	430.84	81.45
42	PR-122 (HS)	133.00	656.24	79.73	172.62	677.40	74.52	152.81	666.82	77.08
43	Pusa Basmati-1401 (HS)	126.77	643.48	80.30	148.89	640.77	76.76	137.83	642.12	78.54
44	Pusa Basmati-1121 (HS)	122.50	688.46	82.21	175.81	714.18	75.38	149.15	701.32	78.73
	Mean	127.42	662.73	80.75	165.77	677.45	75.55	146.60	670.09	78.12
	Overall Mean	52.40	316.75	86.97	56.85	324.01	86.36	54.63	320.38	86.66
Source of Variation		*LSD*	*SE*	Significance	*LSD*	*SE*	Significance	*LSD*	*SE*	Significance
Treatment		1.57	0.78	***	1.61	0.81	***	1.13	0.58	***
Varieties		7.39	3.47	***	7.56	3.82	***	5.31	2.71	***
Year								1.13	0.58	***
Treatment X Varieties		10.47	5.28	***	10.70	5.42	***	7.51	3.82	***
Treatment X Year								1.61	0.81	*
Varieties X Year								7.51	3.82	***
Treatment X Varieties X Year								10.63	5.40	***

*LSD* least significant difference at 0.05%, Significance = *P* < 0.05 (*), *P* < 0.01 (**), *P* < 0.001 (***), *SE* standard error, *R* resistant, *MR* moderately resistant, *S* Susceptible, *HS* highly susceptible.

In 2020 cropping season, unprotected plots recorded higher mean AUDPC values (324.01) compared to the protected plots (56.85), resulting in 86.36% reduction in AUDPC due to the application of fungicide. AUDPC in unprotected varieties ranged from 46.83 to 714.18. The highest AUDPC value was recorded in Pusa Basmati-1121 (714.18) followed by PR-122 (677.40), Pusa Basmati-1401 (640.77), Basmati-129 (595.70), PR-129 (586.18), IR-1460 (585.20), and Pusa Basmati-1612 (532.02). Whereas the lowest AUDPC values were recorded in Basmati-123 (46.83) followed by Pusa-44 (47.34), SJR-5 (57.13), K-448 (57.67), and K-39 (58.82) (Table 2).

Table 2 summarizes the AUDPC values in different rice varieties during 2019 and 2020. The differences in AUDPC values were mainly attributed to the interaction effects among treatment, varieties and year (*P* < 0.001). Unprotected plots had a mean AUDPC values of 320.38, while protected plots had 54.63, representing 86.66% reduction of AUDPC. Compared to unprotected treatments, AUDPC values of highly susceptible, susceptible, moderately resistant and resistant varieties were reduced by 77.08–78.73%, 70.98–92.67%, 75.90–99.47%, and 100%, respectively.

### Infection rate ('r')

In 2019 infection rate ('r') in the protected varieties ranged between 0.00 and 0.206 unit/per day, with the highest ‘r’ values in variety CR-212 (0.206) followed by PR-128 (0.145), PR-114 (0.117), Arize 6444 Gold (0.099), and PR-118 (0.099). In the case of protected varieties ‘r’ ranges between 0.086 and 0.202, with the highest ‘r’ values in variety Pusa Basmati-1121 (0.202) followed by Ranbir-Basmati (0.199), IR-1460 (0.199), Pusa Basmati-1401 (0.195), PR-122 (0.194), and Pusa Basmati-1728 (0.189). Among varieties mean ‘r’ values were maximum in highly susceptible (0.197) varieties followed by susceptible (0.158), moderately resistant (0.113) and resistant (0.104) varieties in unprotected treatment (Table 3).

**Table 3:** Data on infection rate ‘r’ of sheath rot (*S. oryzae*) in different rice varieties during *Kharif* 2019 and 2020.

S. No	Genotypes	Infection rate (r)					
		2019		2020		Mean	
		Protected	Un-Protected	Protected	Un-Protected	Protected	Un-Protected
1	K-448 (R)	0.000	0.119	0.000	0.096	0.000	0.109
2	Basmati-123 (R)	0.000	0.086	0.000	0.101	0.000	0.094
3	K-39 (R)	0.000	0.103	0.000	0.090	0.000	0.097
4	SJR-5 (R)	0.000	0.112	0.000	0.098	0.000	0.105
5	Pusa-44 (R)	0.000	0.100	0.000	0.113	0.000	0.107
	Mean	0.000	0.104	0.000	0.100	0.000	0.102
6	Sanwal-Basmati (MR)	0.073	0.102	0.061	0.088	0.067	0.095
7	Basmati-386 (MR)	0.003	0.099	0.034	0.105	0.020	0.102
8	K-343 (MR)	0.063	0.123	0.116	0.098	0.030	0.111
9	PR-118 (MR)	0.099	0.136	0.113	0.134	0.106	0.135
10	CR-212 (MR)	0.206	0.089	0.222	0.090	0.215	0.089
11	PR-126 (MR)	0.000	0.127	0.082	0.140	0.035	0.134
12	Fateh (MR)	0.003	0.099	0.013	0.121	0.009	0.111
13	PR-121 (MR)	0.013	0.098	0.029	0.103	0.022	0.100
14	SJR-51 (MR)	0.062	0.109	0.072	0.104	0.067	0.106
15	Peeli Pusa (MR)	0.081	0.132	0.125	0.141	0.106	0.137
16	CR-212 (MR)	0.122	0.108	0.100	0.114	0.113	0.111
17	Jaya (MR)	0.077	0.121	0.083	0.116	0.080	0.118
18	Punjab Basmati-1 (MR)	0.035	0.121	0.051	0.131	0.043	0.126
19	Giza-14 (MR)	0.061	0.090	0.098	0.094	0.083	0.092
20	Pusa-Sugandha (MR)	0.047	0.118	0.098	0.126	0.077	0.122
21	CSR-30 (MR)	0.075	0.144	0.124	0.168	0.104	0.157
	Mean	0.064	0.113	0.089	0.117	0.074	0.115
22	Pusa Basmati-1612 (S)	0.051	0.144	0.064	0.147	0.058	0.145
23	Ranbir-Basmati (S)	0.082	0.199	0.088	0.202	0.085	0.201
24	Pusa Basmati-1509 (S)	0.068	0.141	0.053	0.138	0.061	0.140
25	Basmati-370 (S)	0.049	0.129	0.033	0.119	0.042	0.124
26	Basmati-129 (S)	0.058	0.155	0.049	0.160	0.054	0.157
27	Arize 6444 Gold (S)	0.099	0.153	0.115	0.165	0.107	0.159
28	PR-114 (S)	0.117	0.143	0.125	0.147	0.121	0.145
29	PR-124 (S)	0.054	0.153	0.041	0.159	0.047	0.156
30	Kohinoor (S)	0.082	0.138	0.072	0.141	0.077	0.139
31	PR-127 (S)	0.073	0.163	0.050	0.154	0.062	0.159
32	432 (S)	0.111	0.147	0.114	0.143	0.113	0.145
33	PR-128 (S)	0.145	0.150	0.129	0.127	0.138	0.139
34	Basmati-564 (S)	0.048	0.131	0.036	0.134	0.042	0.133
35	PC-19 (S)	0.031	0.149	0.038	0.156	0.035	0.152
36	PR-129 (S)	0.037	0.176	0.053	0.182	0.046	0.179
37	PR-111 (S)	0.027	0.154	0.027	0.150	0.027	0.152
38	PR-113 (S)	0.052	0.179	0.061	0.184	0.057	0.182
39	Ratna (S)	0.051	0.173	0.041	0.166	0.046	0.169
40	Pusa Basmati-1728 (S)	0.044	0.189	0.053	0.196	0.048	0.193
41	IR-1460 (S)	0.044	0.199	0.067	0.204	0.056	0.202
	Mean	0.066	0.158	0.065	0.159	0.066	0.159
42	PR-122 (HS)	0.036	0.194	0.049	0.203	0.043	0.199
43	Pusa Basmati-1401 (HS)	0.052	0.195	0.065	0.204	0.059	0.200
44	Pusa Basmati-1121 (HS)	0.063	0.202	0.082	0.202	0.073	0.202
	Mean	0.050	0.197	0.065	0.203	0.058	0.200
	Overall Mean	0.057	0.138	0.066	0.140	0.061	0.139

In 2020 infection rate (r) in the unprotected varieties ranged between 0.00 and 0.222 unit/per day, with the highest ‘r’ values in variety CR-212 (0.222) followed by PR-128 (0.129), PR-114 (0.125), Peeli Pusa (0.125), and CSR-30 (0.124). In the case of protected varieties ‘r’ ranges between 0.088 and 0.204, with the highest ‘r’ values in variety Pusa Basmati-1401 (0.204) and IR-1460 (0.204), followed by PR-122 (0.203), Pusa Basmati-1121 (0.202), Ranbir-Basmati (0.202), and Pusa Basmati-1728 (0.196). Among varieties, the mean r’ value was maximum in Highly susceptible (0.203) varieties followed by susceptible (0.159), moderately resistant (0.117) and resistant (0.100) varieties in unprotected treatment (Table 3).

Based on the pooled data of the years 2019 and 2020 it was observed that ‘r’ value was less in protected plots, among Highly susceptible (0.058) varieties followed by susceptible (0.066), moderately resistant (0.074) varieties as compared to unprotected plots with ‘r’ values of 0.200, 0.159, and 0.115, respectively in these varieties (Table 3).

### Effect of fungicidal applications on thousand grain weight (TGW)

There was a significant increase in grain yield in all the varieties in protected plots over the mean grain weight of unprotected plots (Table 4; Fig. 1). In protected plots there was substantial increase in grain yield, with a mean TGW of 26.21 and 26.17 g compared to 16.16 and 15.16 g in the unprotected plots during 2019 and 2020, respectively (Table 4; Fig. 1).

**Table 4:** Effect of disease severity of sheath rot on 1000 grain weight (TGW) of different rice varieties during *Kharif* 2019 and 2020.

S. No	Varieties	1000 grain weight (TGW) during *Kharif* (Rainy season)								
		2019			2020			Mean		
		Protected	Un-Protected	TGW loss (%)	Protected	Un-Protected	TGW loss (%)	Protected	Un-Protected	TGW loss (%)
1	K-448 (R)	23.81	23.53	1.18	23.41	23.24	0.73	23.61	23.39	0.96
2	Basmati-123 (R)	24.61	24.54	0.28	24.16	24.12	0.17	24.39	24.33	0.23
3	K-39 (R)	24.00	23.88	0.50	23.63	23.44	0.80	23.82	23.66	0.65
4	SJR-5 (R)	26.02	25.98	0.15	26.55	26.42	0.49	26.29	26.20	0.32
5	Pusa-44 (R)	23.19	23.00	0.82	23.78	23.68	0.42	23.49	23.34	0.62
	Mean	24.33	24.19	0.59	24.31	24.18	0.52	24.32	24.18	0.56
6	Sanwal-Basmati (MR)	23.82	22.12	7.14	24.18	22.96	5.05	24.00	22.54	6.10
7	Basmati-386 (MR)	20.22	18.40	9.00	19.79	18.00	9.04	20.01	18.20	9.02
8	K-343 (MR)	25.02	24.34	2.72	24.63	24.12	2.07	24.83	24.23	2.40
9	PR-118 (MR)	24.81	20.11	18.94	24.25	19.62	19.09	24.53	19.87	19.02
10	CR-212 (MR)	24.83	24.40	1.73	24.64	24.27	1.50	24.74	24.34	1.62
11	PR-126 (MR)	21.80	21.54	1.19	21.56	21.05	2.37	21.68	21.30	1.78
12	Fateh (MR)	25.21	24.86	1.39	25.23	24.54	2.73	25.22	24.70	2.06
13	PR-121 (MR)	25.78	25.60	0.70	26.38	26.00	1.44	26.08	25.80	1.07
14	SJR-51 (MR)	25.59	23.16	9.50	25.34	23.47	7.38	25.47	23.32	8.44
15	Peeli Pusa (MR)	26.86	24.72	7.97	27.61	25.81	6.52	27.24	25.27	7.25
16	CR-212 (MR)	25.78	25.47	1.20	25.00	24.50	2.00	25.39	24.99	1.60
17	Jaya (MR)	25.77	25.14	2.44	24.74	23.89	3.44	25.26	24.52	2.94
18	Punjab Basmati-1 (MR)	25.87	21.48	16.97	25.67	21.84	14.92	25.77	21.66	15.95
19	Giza-14 (MR)	26.20	25.82	1.45	27.10	26.26	3.10	26.65	26.04	2.28
20	Pusa-Sugandha (MR)	23.66	21.26	10.14	23.86	21.01	11.94	23.76	21.14	11.04
21	CSR-30 (MR)	22.62	20.12	11.05	22.40	20.95	6.47	22.51	20.54	8.76
	Mean	24.62	23.03	6.47	24.52	23.02	6.19	24.57	23.03	6.33
22	Pusa Basmati-1612 (S)	28.60	24.60	13.99	29.58	25.60	13.46	29.09	25.10	13.73
23	Ranbir-Basmati (S)	22.61	17.46	22.78	23.00	17.83	22.48	22.81	17.65	22.63
24	Pusa Basmati-1509 (S)	27.66	19.00	31.31	27.20	19.56	28.09	27.43	19.28	29.70
25	Basmati-370 (S)	22.04	17.51	20.55	22.27	19.82	11.00	22.16	18.67	15.78
26	Basmati-129 (S)	23.46	14.98	36.15	23.56	16.40	30.39	23.51	15.69	33.27
27	Arize 6444 Gold (S)	23.00	19.76	14.09	23.22	18.92	18.52	23.11	19.34	16.31
28	PR-114 (S)	25.25	22.53	10.77	24.97	22.01	11.85	25.11	22.27	11.31
29	PR-124 (S)	27.02	21.36	20.95	27.58	22.78	17.40	27.30	..22.07	19.18
30	Kohinoor (S)	25.21	19.65	22.05	24.61	18.83	23.49	24.91	19.24	22.77
31	PR-127 (S)	23.43	16.33	30.30	22.80	17.42	23.60	23.12	16.88	26.95
32	432 (S)	22.06	16.22	26.47	21.40	17.06	20.28	21.73	16.64	23.38
33	PR-128 (S)	28.23	21.26	24.69	28.77	23.01	20.02	28.50	22.14	22.36
34	Basmati-564 (S)	23.18	17.24	25.63	22.80	18.43	19.17	22.99	17.84	22.40
35	PC-19 (S)	21.83	16.74	23.32	21.60	17.12	20.74	21.72	16.93	22.03
36	PR-129 (S)	22.61	13.46	40.47	23.05	15.33	33.49	22.83	14.40	36.98
37	PR-111 (S)	25.60	18.36	28.28	25.01	17.61	29.59	25.31	17.99	28.94
38	PR-113 (S)	27.56	17.22	37.52	28.84	18.72	35.09	28.20	17.97	36.31
39	Ratna (S)	25.63	20.12	21.50	25.97	21.28	18.06	25.80	20.70	19.78
40	Pusa Basmati-1728 (S)	26.61	16.22	39.05	25.83	15.08	41.62	26.22	15.65	40.34
41	IR-1460 (S)	26.06	13.52	48.12	25.62	12.67	50.55	25.84	13.10	49.34
	Mean	24.88	18.18	26.90	24.88	18.77	24.44	24.88	18.48	25.67
42	PR-122 (HS)	22.59	14.44	36.08	22.17	13.37	39.69	22.38	13.91	37.89
43	Pusa Basmati-1401 (HS)	28.23	19.72	30.15	28.78	17.36	39.68	28.51	18.54	34.92
44	Pusa Basmati-1121 (HS)	27.81	14.31	48.52	27.57	14.76	46.47	27.69	14.54	47.51
	Mean	26.21	16.16	38.25	26.17	15.16	41.95	26.19	15.66	40.11
	Overall Mean	24.81	20.49	17.25	24.78	20.69	16.28	24.80	20.59	16.77
Source of Variation		*LSD*	*SE*	Significance	*LSD*	*SE*	Significance	*LSD*	*SE*	Significance
Treatment		0.22	0.07	***	0.14	0.05	***	0.13	0.04	***
Varieties		1.03	0.38	***	0.67	0.24	***	0.62	0.22	***
Year								NA	0.04	NS
Treatment X Varieties		1.50	0.522	***	0.95	0.34	***	0.87	0.31	***
Treatment X Year								NA	0.06	NS
Varieties X Year								0.87	0.31	***
Treatment X Varieties X Year								NA	0.44	NS

*R* resistant, *MR* moderately resistant, *S* susceptible, *HS* highly susceptible.

The differences in TGW between protected and unprotected treatments, varieties and their interaction effects were highly significant (*P* < 0.001) during the cropping season of 2019 and 2020. During these cropping seasons, TGW loss across various varieties varied from 0.15–48.52% to 0.17–50.55% in 2019 and 2020, respectively corresponding to variation in disease severity in different varieties. The highest TGW loss during 2019 and 2020 was recorded in variety Pusa Basmati-1121 (48.52 and 46.47%) followed by IR-1460 (48.12 and 50.55%), PR-129 (40.47 and 33.49%), Pusa Basmati-1728 (39.05 and 41.62%), PR-113 (37.52 and 35.09%), and PR-122 (36.08 and 39.69%).

The resistant varieties recorded lower TGW losses i.e. ranging between 0.15% in SJR-5 to 1.18% in K-448 during 2019 and 0.17% in Basmati-123 to 0.80% in K-39 during 2020, respectively. TGW was less in unprotected than the protected treatments ranging between 13.46–25.98 g and 13.37–26.42 g, during 2019, and 2020, respectively (Table 4). The maximum grain weight among un-protected varieties was observed in SJR-5 (25.98 g), followed by Giza-14 (25.82 g), PR-121 (25.60 g), CR-212 (25.47 g), and Jaya (25.14 g) during 2019 and SJR 5 (26.42 g), followed by Giza-14 (26.26 g), PR 121 (26.00 g), CR 212 (24.50 g), and Jaya (23.89 g) in 2020.

The differences in TGW were mainly attributed to the interaction effects among treatment and varieties and between varieties and years (*P* < 0.001). Protected plots had a mean TGW of 24.80 g compared with 20.59 g in unprotected plots indicating a reduction of 16.77%. Compared to the protected treatments, TGW in varieties IR-1460, Pusa Basmati-1121, Pusa Basmati-1728, PR-122, PR-129, PR-113, Pusa Basmati-1401, and Basmati-129 was reduced by 49.34, 47.51, 40.34, 37.89, 36.98, 36.31, 34.92, and 33.27%, respectively.

### Correlation analysis between disease severity and TGW loss

The correlation coefficient (r) of disease severity with TGW loss was analyzed (Table 5). A strong positive correlation was observed between disease severity and TGW loss across varieties during both the cropping seasons. The correlation coefficient values for disease severity were 0.94 in 2019 and 0.93 in 2020, and 0.94 (mean of both the years).

**Table 5:** Model for the prediction of 1000 grain weight (TGW) loss due to sheath rot severity during cropping season 2019, 2020 and their mean over the years.

Years	r	Regression equation	R^2^	Adjusted R^2^	*SE*	*T* value	*F* value	*P* value	CI^L^	CI^U^	MAPE
2019	0.94***	Y = − 5.65 + 0.91X	0.88	0.87	0.055	16.31	301.7 (1.42)	< *0.001*	20.72	29.75	1.034
2020	0.93***	Y = − 5.87–0.86X	0.87	0.86	0..053	15.80	276.5 (1.42)	< *0.001*	21.05	30.30	1.037
Mean over the year	0.94***	Y = − 5.80–0.89X	0.88	0.88	0.051	16.79	320.5 (1.42)	< *0.001*	20.90	30.01	0.951

*r* correlation coefficient, *SE* standard error, *CI*^*L*^ lower confidence interval, *CI*^*U*^ upper confidence interval, *MAPE* mean absolute percent error.

Significant values are in italic. *** = *P* *value* *<* *0.001*

### Development of predictive model

Linear Regression (LR) models were highly significant (*P* < 0.001) in predicting the TGW loss due to disease severity during both cropping seasons of 2019, 2020 as well as for the combined data over the years.

**Fig. 3:**
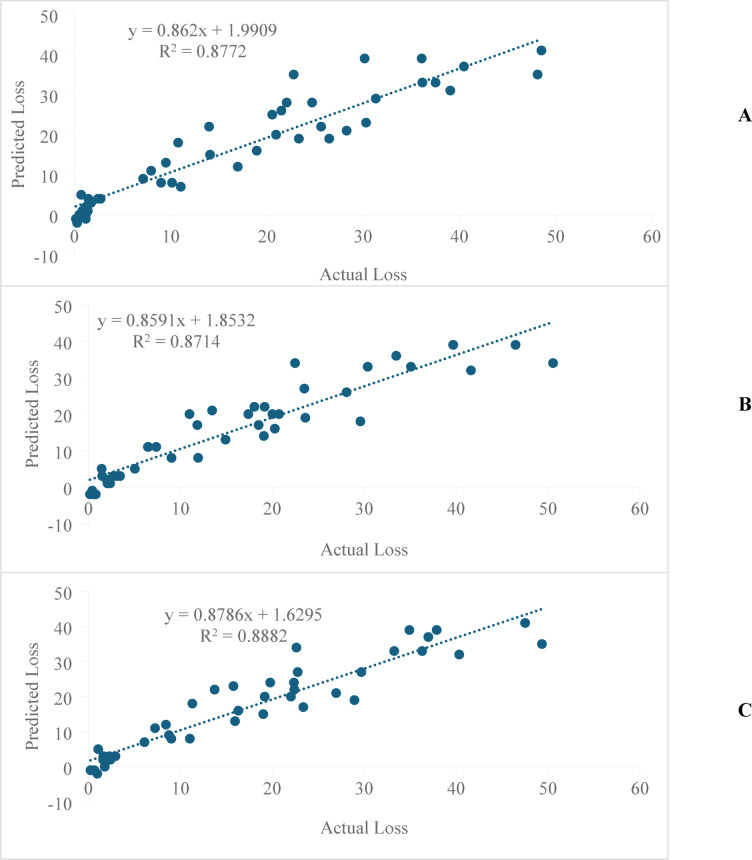
Performance of predicted model for 1000 grain weight (TGW) loss due to disease severity of sheath rot of rice during (**A**) 2019; (**B**) 2020; (**C**) pooled data over the years.

During 2019, the model was highly significant, having F (1, 42) = 301.7, in predicting TGW loss with higher R^2^ (0.88) and adjusted R^2^ (0.87), which depicted that the effect of disease severity had 88% variation in the TGW loss. Model predicted a decrease/increase in per cent loss in TGW by 0.91% corresponding to per unit decrease/increase in disease severity i.e. 95% confidence interval (CI) form 20.72–29.75 (Table 5). Observed versus predicted TGW loss values showed good association i.e. of R^2^ = 0.87 for disease severity (Fig. 3A). The MAPE value of disease severity i.e. 1.034 indicated that the developed model was effective in predicting a loss in TGW due to sheath rot (Table 5).

During 2020, the model was highly significant, having F (1, 42) = 276.5, in predicting loss in TGW with higher R^2^ (0.87) and adjusted R^2^ (0.86), which depicted that the effect of disease severity had 87% variation in the TGW loss. Model predicted a decrease/increase in TGW loss by 0.86% for per unit decrease/increase in disease severity i.e. 95% CI form 21.05–30.30 (Table 5). Observed versus predicted TGW loss values showed good association (R^2^ = 0.87) for disease severity (Fig. 3B). The MAPE value of disease severity i.e. 1.037 (Table 5) indicated that the developed model was effective in predicting loss in TGW due to sheath rot.

For the pooled data i.e. mean of 2019 and 2020, the model was again highly significant for disease severity, with F (1, 42) = 320.5. The R^2^ and adjusted R^2^ values were 0.88, and 0.88, respectively, indicating that disease severity explained 88% of the variation in TGW loss. The present model predicted a decrease/increase in TGW loss by 0.89% per unit decrease/increase in disease severity, with 95% CI of 20.90–30.01% for disease severity (Table 5). The observed versus predicted loss in TGW values showed good association (R^2^ = 0.88) for disease severity (Fig. 3C). The MAPE values of disease severity was 0.951 inferred that the developed model was effective in predicting TGW loss due to sheath rot (Table 5).

## Discussion

The present study showed that disease severity, AUDPC values, infection rate and TGW varied among rice varieties under protected and unprotected conditions. The differences in disease severity, AUDPC values and TGW were mainly attributed to the varieties, treatments, years and interaction effects of treatment and varieties & between varieties and years (*P* < 0.001). In general, higher yield losses were observed in highly susceptible and susceptible varieties infected with sheath rot.

The effect of sheath rot on TGW of rice under artificially inoculated conditions was demonstrated for the first time. The results confirmed the high potential loss caused by sheath rot in rice from different areas globally and verified the findings of earlier workers showing a negative effect of sheath rot on the grain yield of rice^4,26–30^. However, in the present studies conducted under artificial inoculation conditions, the TGW loss was quite high and reached up to 49.34% in the susceptible varieties. This was probably because of the favorable environmental conditions of high morning relative humidity (> 75%), minimum temperature (11.00–20.00 °C) and availability of large amounts of virulent and aggressive inoculum as was confirmed by Mehta et al.^1^. The lesions produced after *S. oryzae* infection may shift the diseased area from a nutrient source (supplying non-structural carbohydrates) to a sink of the pathogen (hijacking nutrients), which resulted in decreased seed setting rate and yield^31,32^. Sheath rot symptoms occurred on flag leaf sheath which is the site from where non-structural carbohydrates translocate to panicle during grain filling stage. Because of the crucial infection position of *S. oryzae*, the hijacking of sugar by *S. oryzae* may directly result in the accumulation of non-structural carbohydrates in the flag leaf sheath leading to poor grain setting^7^.

It was observed that mean disease severity, AUDPC value and infection rate were higher in unprotected plots as compared to protected plots in resistant (4.49%, 51.70, and 0.102), moderately resistant (14.47%, 200.71, and 0.115), susceptible (35.59%, 430.84, and 0.159) and highly susceptible varieties (51.52%, 670.09, and 0.200), respectively. Correspondingly, it led to mean percent reduction in disease severity and AUDPC values of 100 and 100%, 85.05 and 90.60%, 77.00 and 81.45%, 73.37 and 78.12% were recorded in resistant, moderately resistant, susceptible and highly susceptible varieties, respectively under protected conditions over the years (Tables 1, 2, and 3; Fig. 1). Under unprotected conditions, the mean TGW was less in resistant (24.18 g), moderately resistant (23.03 g), susceptible (18.48 g) and highly susceptible varieties (15.66 g) as compared to 24.32, 24.57, 24.88, and 26.19 g under protected conditions (Table 4; Fig. 1). Integrated use of resistant varieties and fungicide applications significantly reduced sheath rot severity and disease progression over the years. The findings contended that using moderately resistant rice varieties integrated with judicious combination of fungicides could reduce sheath rot damage and yield losses and increase yield gains under high disease pressure. Moreover, this practice will lead to reduced use of fungicides thus decreased soil, water and air pollution.

Despite the fact that efficacy of foliar fungicides largely depends on various factors viz. the level of host resistance, the efficacy of the fungicide, foliar coverage achieved, disease pressure, and weather conditions^33^. A significant reduction in disease severity could be due to the suppressive roles of the fungicide along with the genetic resistant background of the varieties to subdue infectivity of the pathogen and lesion development. Integration of fungicide and host resistance could also decrease germination, infection, growth and spread of the pathogens. As has been observed in the present study, the efficacy of strobilurins (azoxystrobin, trifloxystrobin, picoxystrobin etc.) and triazoles (difenoconazole, epoxiconazole, hexaconazole, propiconazole, tebuconazole etc.) fungicides was also reported against multiple diseases viz. blast, brown spots, grain discoloration, sheath rot, dirty panicle and sheath blight of rice^34–40^.

It was observed that TGW loss had significant and positive correlation with disease severity. Present results are in conformity with the findings of Chuwa et al.^41^ who assessed loss in grain yield due to blast disease of rice and emphasized that leaf and panicle rice blast disease severities were significantly and positively correlated with grain yield losses. Similarly, Onwunali and Mabagala^42^ reported loss in grain yield and 1000 grain weight in five maize varieties due to northern corn leaf blight disease. Moreover, Lore et al.^43^ observed that disease variables related to sheath blight were positively correlated with yield loss in rice. Mengesha et al.^10^ also reported loss in grain yield of pea due to Ascochyta blight disease.

As has been observed in the present study, prediction of losses in grain yield using linear regression analysis (LRA) was reported in ascochyta blight in pea^10^, northern maize leaf blight of maize^42^, sheath blight of rice^43^, anthracnose of sorghum^44^, spot blotch of wheat^45^, southern maize leaf blight^46^, maize fall army worm^47^.

Mean absolute percent error (MAPE) is a very useful parameter for assessing the accuracy and effectiveness of performed forecasts. In the present studies, MAPE achieved low values (< 10%) during the years 2019, 2020 and the pooled data over the years, which is in agreement with the findings of Peng et al.^24^, who revealed that the degree of goodness-of-fit of the model was perfect thus it enabled great possibilities of its application. Additionally, the MAPE values are frequently used to assess the usefulness of forecast models^23,48,49^. Observed verses predicted plot also depicted a good association during 2019, 2020 and for their mean pooled over the years. In the present study, it was observed that sheath rot significantly reduced rice grain weight in all the susceptible cultivars, while the yield loss in resistant cultivars was not reduced significantly and to large extent. Integrated use of host resistance and fungicide applications slowed down disease progression and disease pressure, minimized relative loss in TGW and enhanced agronomic performances of rice varieties. Application of fungicide azoxystrobin (11%) + tebuconazole (13.8%) SC significantly reduced sheath rot severity and comparably minimized loss in TGW, irrespective of the resistance status of the varieties.

## Conclusions

The study revealed that azoxystrobin (11%) + tebuconazole (13.8%) SC fungicide sprays against sheath rot disease enhanced TGW of the rice varieties with highly significant impact in susceptible and highly susceptible varieties. Cultivation of such susceptible varieties should be avoided in disease prone areas and in case no alternative is available crop must be adequately scouted for sheath rot, and initiation of azoxystrobin (11%) + tebuconazole (13.8%) SC sprays must be routinely timed prior to or as soon the disease is detected in the field. The predictive model for TGW loss indicated good fitness with strong and reliable validity. Such models can be used to estimate potential losses of rice caused by sheath rot disease. Future research should focus on the integration of agronomic practices viz. sowing date, field hygiene, clean seed, crop rotation practices, host resistance, seed treatment and judicious alternate application of fungicides to ensure profitable and eco-friendly rice production.

## Electronic supplementary material

Below is the link to the electronic supplementary material.

Supplementary Material 1

## Data Availability

The data that support the findings of this study are available from the corresponding author upon reasonable request.
