# Supplementary material for: Determination of yield losses against sheath rot caused by Sarocladium oryzae in rice varieties with differential resistance
Source: Sci Rep. 2025 Oct 17;15:36309. doi: 10.1038/s41598-025-05104-y (PMC12534572; doi:10.1038/s41598-025-05104-y)
Supplement: Supplementary file 1 — Supplementary Material 1 [file 41598_2025_5104_MOESM1_ESM.docx]

**Supplementary Table 1.** Information on the test varieties, their pedigree, origin and field reaction to sheath rot.

| **Varieties** | **Pedigree** | **Origin** | **Field Reaction to sheath rot** | **Remarks** |
| --- | --- | --- | --- | --- |
| K-448 | - | SKUAST-K | R | Non-Basmati |
| Basmati-123 | - | - | R | Basmati |
| K-39 | - | SKUAST-K | R | Non-Basmati |
| SJR-5 | IR 25393-57 / RD 23 // IR 27316-96 /// SPRLR 77205 -3-2 / SPRLR 79134-51-2 | SKUAST-J | R | Non-Basmati |
| Pusa-44 | IARI-5901-2 x IR-8 | IARI | R | Non-Basmati |
| Sanwal-Basmati | - | - | MR | Basmati |
| Basmati-386 | Selection from Pak. Basmati | PAU | MR | Basmati |
| K-343 | - | SKUAST-K | MR | Non-Basmati |
| PR-118 | Pusa 44/PR 110// Pusa 44*3 | PAU | MR | Non-Basmati |
| CR-212 | - | - | MR | Non-Basmati |
| PR-126 | PR 122 / IR64 | PAU | MR | Non-Basmati |
| Fateh | - | - | MR | Non-Basmati |
| PR-121 | PR 116///PR 108/ IRRI 76//PR 106-P2 | PAU | MR | Non-Basmati |
| SJR-51 | - | SKUAST-J | MR | Non-Basmati |
| Peeli Pusa | - | - | MR | Non-Basmati |
| CR-212 | - | - | MR | Non-Basmati |
| Jaya | TN 1 x Type 141 | - | MR | Non-Basmati |
| Punjab Basmati-1 | Sona /Basmati 370 | PAU | MR | Basmati |
| Giza-14 | - | SKUAST-J | MR | Non-Basmati |
| Pusa-Sugandha | - | - | MR | Basmati |
| CSR-30 | BR4-10/ Basmati 386 (Pak basmati) | HR | MR | Basmati |
| Pusa Basmati-1612 | - | - | S | Basmati |
| Ranbir-Basmati | Pure line selection from basmati 370-90-95 | SKUAST-J | S | Basmati |
| Pusa Basmati-1509 | Pusa1301/Pusa1121 | IARI | S | Basmati |
| Basmati-370 | Pure line selection Dehraduni Basmati landrace | SKUAST-J | S | Basmati |
| Basmati-129 | - | - | S | Basmati |
| Arize 6444 Gold | - | Bayer Crop Sci. | S | Non-Basmati |
| PR-114 | TN1/Patong 32//PR 106*4///IR8 | PAU | S | Non-Basmati |
| PR-124 | - | PAU | S | Non-Basmati |
| Kohinoor | - | - | S | Non-Basmati |
| PR-127 | Pusa44/O.glaberrima (102 600 b)// Pusa 44*3 | PAU | S | Non-Basmati |
| 432 | - | - | S | Non-Basmati |
| PR-128 | PAU 201/PAU 3699-13-2-3-1//PAU 201 | PAU | S | Non-Basmati |
| Basmati-564 | - | - | S | Basmati |
| PC-19 | - | - | S | Non-Basmati |
| PR-129 | PAU 201/PAU 3699-13-2-3-1//PAU 201 | PAU | S | Non-Basmati |
| PR-111 | IR 54/PR 106 | PAU | S | Non-Basmati |
| PR-113 | IR 8// RP 2151-173-1 8/IR8*4 | PAU | S | Non-Basmati |
| Ratna | TKM-6 x IR-8 | - | S | Non-Basmati |
| Pusa Basmati-1728 | - | IARI | S | Basmati |
| IR-1460 | - | - | S | Basmati |
| PR-122 (HS) | PR 108 / IRRI-76//PR 106-P1 | PAU | HS | Non-Basmati |
| Pusa Basmati-1401 (HS) | Pusa 1401-97-7-1-4 (IET 18005) | IARI | HS | Basmati |
| Pusa Basmati-1121 (HS) | Pusa 614-1-2/ Pusa 614-2-4 3 | IARI | HS | Basmati |

R= resistant; MR= moderately resistant; S= susceptible; HS= highly susceptible, - = not available

**Supplementary Table 2** Weather parameters during 2019 cropping season.

| Date | Maximum Temperature (°C) | | Minimum Temperature (°C) | | Rainfall | Relative Humidity (%) | |
| --- | --- | --- | --- | --- | --- | --- | --- |
|  | Actual | Normal | Actual | Normal |  | Morning | Evening |
| 30-Jul-19 | 34.8 | 32.9 | 27.5 | 24.5 | 0 | 88 | 63 |
| 31-Jul-19 | 28.8 | 32.9 | 24.4 | 24.9 | 43 | 98 | 80 |
| 1-Aug-19 | 28 | 33.3 | 25.9 | 24.5 | 0 | 86 | 87 |
| 2-Aug-19 | 31.8 | 33.5 | 25.1 | 24.4 | 0 | 92 | 67 |
| 3-Aug-19 | 34.2 | 33.1 | 26.4 | 24.2 | 0 | 75 | 58 |
| 4-Aug-19 | 35.4 | 33.3 | 27.2 | 24 | 0 | 82 | 54 |
| 5-Aug-19 | 35.6 | 33.6 | 26.9 | 23.9 | 0 | 81 | 65 |
| 6-Aug-19 | 34.6 | 32.2 | 26.6 | 23.9 | 0 | 84 | 67 |
| 7-Aug-19 | 34.6 | 33.2 | 24.8 | 23.9 | 10.8 | 93 | 74 |
| 8-Aug-19 | 35.6 | 33.2 | 27 | 24.2 | 0 | 83 | 55 |
| 9-Aug-19 | 30.6 | 33.8 | 29.4 | 24.4 | 0 | 85 | 97 |
| 10-Aug-19 | 33.4 | 33.4 | 26.6 | 24.7 | 12.2 | 92 | 67 |
| 11-Aug-19 | 34.6 | 33.8 | 28 | 24.6 | 0 | 89 | 64 |
| 12-Aug-19 | 35.2 | 32.8 | 28 | 24.4 | 0 | 85 | 66 |
| 13-Aug-19 | 31.6 | 33 | 23.1 | 23.9 | 78 | 98 | 69 |
| 14-Aug-19 | 32.8 | 32.9 | 24.9 | 24.3 | 1.8 | 95 | 70 |
| 15-Aug-19 | 35 | 33.2 | 26.4 | 24.4 | 3 | 85 | 65 |
| 16-Aug-19 | 32.5 | 33.2 | 25.4 | 24 | 0 | 89 | 69 |
| 17-Aug-19 | 26.5 | 33.9 | 24.2 | 24.3 | 2.5 | 90 | 98 |
| 18-Aug-19 | 32.6 | 33.6 | 23 | 24.3 | 44.8 | 90 | 62 |
| 19-Aug-19 | 33 | 33.5 | 21.2 | 24.4 | 1 | 79 | 60 |
| 20-Aug-19 | 33.4 | 33.5 | 25.2 | 24.3 | 0 | 89 | 63 |
| 21-Aug-19 | 34.8 | 33.6 | 25.8 | 24.2 | 0 | 86 | 55 |
| 22-Aug-19 | 35.4 | 33.3 | 26.4 | 24.3 | 0 | 85 | 57 |
| 23-Aug-19 | 34.8 | 33.2 | 25.6 | 23.9 | 0 | 75 | 61 |
| 24-Aug-19 | 35.8 | 32.9 | 29 | 23.5 | 0 | 89 | 53 |
| 25-Aug-19 | 33.8 | 33.5 | 25.5 | 23.4 | 0 | 77 | 61 |
| 26-Aug-19 | - | 33.7 | 24 | 23.6 | 0 | 90 | 59 |
| 27-Aug-19 | 35.4 | 33.1 | 27.5 | 23.7 | 0 | 89 | 64 |
| 28-Aug-19 | 36.6 | 33.2 | 27.6 | 23.5 | 0 | 97 | 51 |
| 29-Aug-19 | 36.6 | 33.4 | 27.8 | 23.6 | 0 | 84 | 52 |
| 30-Aug-19 | 32.6 | 33.9 | 24 | 23.6 | 2 | 88 | 63 |
| 31-Aug-19 | 35.4 | 33.7 | 26.4 | 23.3 | 0 | 83 | 61 |
| 1-Sep-19 | 35.2 | 33.7 | 28 | 23.6 | 0 | 88 | 77 |
| 2-Sep-19 | 35.5 | 33.2 | 25.4 | 23.5 | 0 | 85 | 79 |
| 3-Sep-19 | 36 | 33 | 27.1 | 23.2 | 0 | 88 | 59 |
| 4-Sep-19 | 35 | 33.5 | 25 | 23.1 | 3 | 83 | 64 |
| 5-Sep-19 | 33 | 33.4 | 23.6 | 23.1 | 4.2 | 90 | 64 |
| 6-Sep-19 | 34.4 | 33.3 | 27.4 | 22.1 | 0 | 92 | 67 |
| 7-Sep-19 | 33.6 | 33.3 | 27 | 22.6 | 0 | 85 | 63 |
| 8-Sep-19 | 35 | 33.5 | 23.4 | 23.1 | 1.8 | 77 | 57 |
| 9-Sep-19 | 35.4 | 32.7 | 25.9 | 22.8 | 0 | 83 | 55 |
| 10-Sep-19 | 36.2 | 33.3 | 27.5 | 22.4 | 0 | 87 | 62 |
| 11-Sep-19 | 36.2 | 33.3 | 28.2 | 22.4 | 0 | 87 | 63 |
| 12-Sep-19 | 36 | 33.3 | 28.2 | 22.1 | 0 | 87 | 64 |
| 13-Sep-19 | 33.4 | 33.2 | 24.4 | 22.4 | 0 | 90 | 79 |
| 14-Sep-19 | 35.2 | 32.8 | 24.6 | 22.4 | 0 | 77 | 56 |
| 15-Sep-19 | 35.2 | 32.6 | 26.2 | 22.1 | 0 | 86 | 62 |
| 16-Sep-19 | 32.2 | 32.6 | 22.2 | 22.2 | 3 | 95 | 60 |
| 17-Sep-19 | 34 | 33 | 23.4 | 22 | 0 | 97 | 60 |
| 18-Sep-19 | 34 | 33.2 | 22.4 | 22.2 | 0 | 76 | 54 |
| 19-Sep-19 | 29.4 | 33.4 | 21.4 | 22.3 | 41.2 | 98 | 76 |
| 20-Sep-19 | 30.4 | 33.4 | 23.6 | 22.4 | 20.2 | 92 | 65 |
| 21-Sep-19 | 32.4 | 33.2 | 22.5 | 22.2 | 0 | 87 | 59 |
| 22-Sep-19 | 33 | 32.1 | 23 | 21.3 | 0 | 88 | 55 |
| 23-Sep-19 | 31.5 | 32.5 | 24.6 | 20.9 | 0 | 88 | 50 |
| 24-Sep-19 | 30.6 | 32.9 | 24.4 | 21 | 0 | 92 | 69 |
| 25-Sep-19 | 32.8 | 32.8 | 24.5 | 20.9 | 0 | 93 | 63 |
| 26-Sep-19 | 32 | 32.9 | 24.9 | 21.1 | 0 | 92 | 67 |
| 27-Sep-19 | 30.4 | 33 | 22.3 | 21 | 1.2 | 95 | 71 |
| 28-Sep-19 | 26.8 | 33 | 23.9 | 20.8 | 0 | 97 | 98 |
| 29-Sep-19 | 25.4 | 32.9 | 21.1 | 20.4 | 58.2 | 90 | 96 |
| 30-Sep-19 | 29.8 | 33 | 20.8 | 20.3 | 34 | 93 | 67 |
| 1-Oct-19 | 29.4 | 33.1 | 17 | 20.1 | 12.8 | 88 | 61 |
| 2-Oct-19 | 30.5 | 33.1 | 19.9 | 20.3 | 0 | 80 | 59 |
| 3-Oct-19 | 29.8 | 32.7 | 21.2 | 20 | 0 | 93 | 77 |
| 4-Oct-19 | 26.5 | 32.4 | 17.6 | 20 | 8.6 | 85 | 75 |
| 5-Oct-19 | 29.5 | 32.6 | 16.8 | 19.6 | 0 | 89 | 58 |
| 6-Oct-19 | 27 | 32.5 | 18.8 | 18.9 | 0 | 91 | 69 |
| 7-Oct-19 | 28.6 | 32.7 | 20.6 | 19.3 | 0 | 94 | 53 |
| 8-Oct-19 | 29.8 | 32.6 | 17.2 | 16.3 | 0 | 77 | 51 |
| 9-Oct-19 | 30.6 | 32.7 | 18.4 | 16 | 0 | 89 | 53 |
| 10-Oct-19 | 29.8 | 32 | 17.4 | 15.7 | 0 | 78 | 54 |
| 11-Oct-19 | 30.6 | 32 | 18.2 | 15.8 | 0 | 91 | 47 |
| 12-Oct-19 | 30.8 | 31.4 | 18.8 | 15.6 | 0 | 89 | 53 |
| 13-Oct-19 | 30.9 | 31.3 | 19.6 | 15.4 | 0 | 88 | 55 |
| 14-Oct-19 | 31.2 | 31.4 | 20.6 | 15.4 | 0 | 93 | 57 |
| 15-Oct-19 | 30.8 | 30.8 | 20 | 16.3 | 0 | 84 | 54 |
| 16-Oct-19 | 30.2 | 30.9 | 20.1 | 16 | 0 | 93 | 56 |
| 17-Oct-19 | 30 | 30.7 | 20.6 | 15.7 | 0 | 93 | 60 |
| 18-Oct-19 | 26.6 | 30.9 | 16.4 | 15.8 | 9.2 | 76 | 45 |
| 19-Oct-19 | 28.7 | 30.7 | 15.4 | 15.6 | 0 | 83 | 51 |
| 20-Oct-19 | 28.6 | 30.1 | 14.4 | 15.4 | 0 | 94 | 48 |
| 21-Oct-19 | 29 | 30.7 | 14.4 | 15.4 | 0 | 88 | 50 |
| 22-Oct-19 | 29 | 30.2 | 13.4 | 15 | 0 | 88 | 42 |
| 23-Oct-19 | 29.5 | 30.2 | 15.4 | 14.8 | 0 | 83 | 48 |
| 24-Oct-19 | 29.6 | 30.2 | 15.6 | 14.5 | 0 | 81 | 47 |
| 25-Oct-19 | 29.4 | 30 | 14.8 | 14.5 | 0 | 83 | 45 |
| 26-Oct-19 | 29 | 29.8 | 14.8 | 14.8 | 0 | 83 | 44 |
| 27-Oct-19 | 29 | 30 | 13.4 | 14.5 | 0 | 88 | 37 |
| 28-Oct-19 | 29.5 | 29.9 | 13.9 | 14.4 | 0 | 86 | 42 |
| 29-Oct-19 | 29.2 | 29.7 | 15.7 | 13.9 | 0 | 88 | 49 |
| 30-Oct-19 | 28.7 | 29.8 | 16 | 14 | 0 | 90 | 50 |
| 31-Oct-19 | 29 | 29.5 | 16.4 | 13.8 | 0 | 90 | 46 |
| 1-Nov-19 | 25.2 | 29.4 | 15.8 | 13.3 | 0 | 90 | 70 |
| 2-Nov-19 | 28.4 | 29.2 | 15 | 13.3 | 0 | 92 | 57 |
| 3-Nov-19 | 27.2 | 28.7 | 18 | 12.8 | 0 | 92 | 54 |
| 4-Nov-19 | 28.2 | 28.6 | 13.4 | 13.1 | 0 | 87 | 50 |
| 5-Nov-19 | 29.6 | 28.3 | 12.6 | 12.5 | 0 | 89 | 48 |
| 6-Nov-19 | 27.5 | 28.5 | 15.8 | 12.3 | 0 | 82 | 39 |
| 7-Nov-19 | 17.4 | 28.1 | 15.5 | 12.2 | 13.6 | 85 | 100 |
| 8-Nov-19 | 24.8 | 27.8 | 11.3 | 12 | 38.2 | 66 | 45 |
| 9-Nov-19 | 27.4 | 27.9 | 11.1 | 11.8 | 0 | 76 | 40 |
| 10-Nov-19 | 26.6 | 27.7 | 12.2 | 11.6 | 0 | 93 | 44 |
| 11-Nov-19 | 23.8 | 27.5 | 12.8 | 11.4 | 0 | 91 | 41 |
| 12-Nov-19 | 24.2 | 27.2 | 14 | 11 | 0 | 86 | 57 |
| 13-Nov-19 | 24.2 | 27.2 | 12.8 | 11.1 | 0 | 96 | 54 |
| 14-Nov-19 | 22.4 | 27.3 | 14.3 | 11 | 0 | 98 | 79 |
| 15-Nov-19 | 23 | 26.9 | 16.2 | 10.7 | 0.4 | 84 | 61 |
| 16-Nov-19 | 24 | 26.5 | 15.4 | 10.2 | 0 | 77 | 73 |
| 17-Nov-19 | 25 | 26.1 | 10.8 | 9.9 | 2.4 | 93 | 55 |
| 18-Nov-19 | 25.2 | 26 | 10.4 | 10.4 | 0 | 95 | 53 |
| 19-Nov-19 | 24.2 | 26.4 | 11.4 | 10.1 | 0 | 93 | 54 |
| 20-Nov-19 | 21.8 | 26 | 11.6 | 9.6 | 0 | 95 | 71 |
| 21-Nov-19 | 20.4 | 25.8 | 13.4 | 9.5 | 0 | 91 | 81 |
| 22-Nov-19 | 18 | 25.5 | 13.6 | 9.4 | 0 | 96 | 86 |
| 23-Nov-19 | 25 | 25.2 | 14.6 | 9.2 | 0.8 | 89 | 49 |
| 24-Nov-19 | 24 | 25.1 | 12.4 | 8.9 | 0 | 91 | 57 |
| 25-Nov-19 | 23.6 | 24.8 | 10 | 8.8 | 0 | 93 | 45 |
| 26-Nov-19 | 23.4 | 24.8 | 10.4 | 8.7 | 0 | 95 | 42 |
| 27-Nov-19 | 20 | 24.3 | 12 | 8 | 0 | 93 | 90 |
| 28-Nov-19 | 20 | 24.1 | 11.8 | 8 | 22 | 95 | 64 |
| 29-Nov-19 | 22.2 | 24 | 11.8 | 7.7 | 0 | 93 | 47 |
| 30-Nov-19 | 23.4 | 23.9 | 8.6 | 7.7 | 0 | 95 | 38 |
| 1-Dec-19 | 22.2 | 24.1 | 7.6 | 7.8 | 0 | 89 | 43 |
| 2-Dec-19 | 22 | 23.7 | 6.6 | 7.7 | 0 | 92 | 40 |

**Supplementary Table 3** Weather parameters during 2020 cropping season.

| Date | Maximum Temperature (°C) | | Minimum Temperature (°C) | | Rainfall | Relative Humidity (%) | |
| --- | --- | --- | --- | --- | --- | --- | --- |
|  | Actual | Normal | Actual | Normal | Actual | Normal | Actual |
| 30-Jul-20 | 31.2 | 32.9 | 24.6 | 24.5 | 1.6 | 88 | 75 |
| 31-Jul-20 | 33.8 | 32.9 | 26.4 | 24.9 | 0 | 89 | 65 |
| 1-Aug-20 | 32.4 | 33.3 | 23 | 24.5 | 33.6 | 98 | 73 |
| 2-Aug-20 | 34.8 | 33.5 | 27.2 | 24.4 | 0 | 82 | 67 |
| 3-Aug-20 | 36.2 | 33.1 | 29 | 24.2 | 0 | 84 | 60 |
| 4-Aug-20 | 36.2 | 33.3 | 22 | 24 | 19.4 | 81 | 55 |
| 5-Aug-20 | 37 | 33.6 | 28 | 23.9 | 0 | 87 | 57 |
| 6-Aug-20 | 37 | 32.2 | 27.8 | 23.9 | 0 | 78 | 57 |
| 7-Aug-20 | 33 | 33.2 | 29 | 23.9 | 0 | 85 | 64 |
| 8-Aug-20 | 35.2 | 33.2 | 27.6 | 24.2 | 2 | 89 | 65 |
| 9-Aug-20 | 34 | 33.8 | 23.4 | 24.4 | 18 | 93 | 75 |
| 10-Aug-20 | 33.4 | 33.4 | 27.6 | 24.7 | 1.2 | 85 | 83 |
| 11-Aug-20 | 34.6 | 33.8 | 25.5 | 24.6 | 4.4 | 90 | 80 |
| 12-Aug-20 | 33 | 32.8 | 24 | 24.6 | 0 | 89 | 75 |
| 13-Aug-20 | 33 | 33 | 24.6 | 23.9 | 93.6 | 94 | 78 |
| 14-Aug-20 | 32.8 | 32.9 | 26 | 24.3 | 9 | 95 | 69 |
| 15-Aug-20 | 35 | 33.2 | 26.5 | 24.4 | 1 | 92 | 64 |
| 16-Aug-20 | 35 | 33.2 | 28 | 24 | 0 | 88 | 63 |
| 17-Aug-20 | 32.4 | 33.9 | 24.4 | 24.3 | 65 | 95 | 79 |
| 18-Aug-20 | 33.8 | 33.6 | 27.4 | 24.3 | 0 | 94 | 72 |
| 19-Aug-20 | 29 | 33.5 | 23.6 | 24.4 | 17 | 96 | 82 |
| 20-Aug-20 | 28.2 | 33.5 | 24.9 | 24.3 | 24 | 98 | 92 |
| 21-Aug-20 | 27.8 | 33.6 | 25.5 | 24.2 | 2.6 | 92 | 80 |
| 22-Aug-20 | 33.8 | 33.3 | 23.4 | 24.3 | 0 | 78 | 58 |
| 23-Aug-20 | 34.4 | 33.2 | 26.4 | 23.9 | 0 | 83 | 63 |
| 24-Aug-20 | 33 | 32.9 | 27.5 | 23.5 | 0 | 89 | 71 |
| 25-Aug-20 | 31.4 | 33.5 | 24.9 | 23.4 | 27.2 | 98 | 72 |
| 26-Aug-20 | 29.9 | 33.7 | 24 | 23.6 | 117 | 100 | 78 |
| 27-Aug-20 | 26.2 | 33.1 | 24.4 | 23.7 | 38.8 | 100 | 98 |
| 28-Aug-20 | 29.9 | 33.2 | 23.2 | 23.5 | 88 | 98 | 74 |
| 29-Aug-20 | 32.6 | 33.4 | 25 | 23.6 | 0 | 90 | 66 |
| 30-Aug-20 | 34 | 33.9 | 26 | 23.6 | 0 | 85 | 61 |
| 31-Aug-20 | 32.6 | 33.7 | 24.2 | 23.3 | 32.4 | 92 | 67 |
| 1-Sep-20 | 32.2 | 33.7 | 25.5 | 23.6 | 1.4 | 83 | 63 |
| 2-Sep-20 | 33.4 | 33.2 | 26 | 23.5 | 0 | 90 | 71 |
| 3-Sep-20 | 33 | 33 | 27.4 | 23.2 | 0 | 89 | 73 |
| 4-Sep-20 | 28.6 | 33.5 | 26 | 23.1 | 0 | 92 | 82 |
| 5-Sep-20 | 32.8 | 33.4 | 24.6 | 23.1 | 0 | 85 | 63 |
| 6-Sep-20 | 33 | 33.3 | 26.8 | 22.1 | 0 | 89 | 57 |
| 7-Sep-20 | 33.6 | 33.3 | 21.6 | 22.6 | 17.6 | 88 | 66 |
| 8-Sep-20 | 33.8 | 33.5 | 25 | 23.1 | 0 | 92 | 51 |
| 9-Sep-20 | 33 | 32.7 | 25 | 22.8 | 0 | 87 | 55 |
| 10-Sep-20 | 35 | 33.3 | 26 | 22.4 | 0 | 89 | 55 |
| 11-Sep-20 | 35 | 33.3 | 25.6 | 22.4 | 0 | 87 | 56 |
| 12-Sep-20 | 35 | 33.3 | 26.2 | 22.1 | 0 | 85 | 58 |
| 13-Sep-20 | 35.2 | 33.2 | 26.4 | 22.4 | 0 | 85 | 52 |
| 14-Sep-20 | 35.4 | 32.8 | 26 | 22.4 | 0 | 87 | 56 |
| 15-Sep-20 | 35.4 | 32.6 | 26 | 22.1 | 0 | 82 | 61 |
| 16-Sep-20 | 35 | 32.6 | 26 | 22.2 | 0 | 82 | 56 |
| 17-Sep-20 | 36 | 32.9 | 26.2 | 22.2 | 0 | 85 | 52 |
| 18-Sep-20 | 36.2 | 33.1 | 25.2 | 22.2 | 0 | 84 | 46 |
| 19-Sep-20 | 35.6 | 33.3 | 25.6 | 22.1 | 0 | 82 | 55 |
| 20-Sep-20 | 36 | 33.4 | 25.4 | 22.1 | 0 | 84 | 47 |
| 21-Sep-20 | 36.4 | 33.4 | 24.8 | 22 | 0 | 78 | 51 |
| 22-Sep-20 | 36 | 33.4 | 24.6 | 20.4 | 0 | 81 | 50 |
| 23-Sep-20 | 36.4 | 32.5 | 25 | 20.6 | 0 | 83 | 54 |
| 24-Sep-20 | 35.2 | 33 | 24.4 | 20.6 | 0 | 83 | 53 |
| 25-Sep-20 | 34.8 | 32.8 | 24.4 | 20.5 | 0 | 80 | 53 |
| 26-Sep-20 | 33.5 | 32.9 | 21 | 20.6 | 0 | 71 | 63 |
| 27-Sep-20 | 34.4 | 33.1 | 20 | 20.7 | 0 | 72 | 47 |
| 28-Sep-20 | 33.8 | 33 | 20 | 20.5 | 0 | 76 | 45 |
| 29-Sep-20 | 33.2 | 32.9 | 20.4 | 20.1 | 0 | 84 | 45 |
| 30-Sep-20 | 34 | 33.1 | 19.4 | 19.8 | 0 | 88 | 39 |
| 1-Oct-20 | 34.2 | 33.1 | 19.8 | 20.1 | 0 | 74 | 38 |
| 2-Oct-20 | 34 | 33 | 19.4 | 20.3 | 0 | 71 | 46 |
| 3-Oct-20 | 34 | 32.7 | 17.8 | 20 | 0 | 73 | 33 |
| 4-Oct-20 | 33.6 | 32.3 | 17.4 | 19.9 | 0 | 79 | 39 |
| 5-Oct-20 | 33.8 | 32.6 | 17.4 | 19.4 | 0 | 82 | 36 |
| 6-Oct-20 | 33.8 | 32.5 | 17.4 | 18.8 | 0 | 77 | 44 |
| 7-Oct-20 | 33.6 | 32.6 | 18 | 19.4 | 0 | 78 | 42 |
| 8-Oct-20 | 33.6 | 32.6 | 19 | 18.8 | 0 | 76 | 41 |
| 9-Oct-20 | 33.8 | 32.7 | 19 | 18.4 | 0 | 81 | 44 |
| 10-Oct-20 | 33.4 | 32 | 19 | 18.3 | 0 | 82 | 47 |
| 11-Oct-20 | 33.6 | 32 | 18 | 17.7 | 0 | 79 | 41 |
| 12-Oct-20 | 33.6 | 31.4 | 17.8 | 17 | 0 | 89 | 38 |
| 13-Oct-20 | 33.2 | 31.3 | 17.4 | 17.3 | 0 | 81 | 39 |
| 14-Oct-20 | 33 | 31.4 | 15.6 | 16.7 | 0 | 82 | 36 |
| 15-Oct-20 | 32.6 | 30.8 | 15 | 16.3 | 0 | 83 | 32 |
| 16-Oct-20 | 32.6 | 30.9 | 14.8 | 16 | 0 | 88 | 33 |
| 17-Oct-20 | 33.4 | 30.7 | 14.4 | 16.7 | 0 | 94 | 30 |
| 18-Oct-20 | 32.6 | 30.9 | 13.8 | 15.8 | 0 | 96 | 31 |
| 19-Oct-20 | 32.2 | 30.7 | 14.2 | 15.6 | 0 | 90 | 33 |
| 20-Oct-20 | 31.6 | 30.1 | 14.4 | 15.4 | 0 | 90 | 31 |
| 21-Oct-20 | 31 | 30.7 | 14 | 15.1 | 0 | 91 | 33 |
| 22-Oct-20 | 30.6 | 30.2 | 12.6 | 15 | 0 | 87 | 31 |
| 23-Oct-20 | 30.6 | 30.2 | 14.4 | 14.8 | 0 | 87 | 37 |
| 24-Oct-20 | 31 | 30.2 | 14 | 14.5 | 0 | 92 | 34 |
| 25-Oct-20 | 30 | 30 | 12.6 | 14.5 | 0 | 93 | 40 |
| 26-Oct-20 | 29.8 | 29.8 | 12 | 14.8 | 0 | 91 | 41 |
| 27-Oct-20 | 29.6 | 30 | 10.6 | 14.5 | 0 | 93 | 40 |
| 28-Oct-20 | 29.5 | 29.9 | 10 | 14.4 | 0 | 92 | 36 |
| 29-Oct-20 | 29 | 29.7 | 10.6 | 13.9 | 0 | 86 | 29 |
| 30-Oct-20 | 28.6 | 29.8 | 11.4 | 14 | 0 | 91 | 28 |
| 31-Oct-20 | 28 | 29.5 | 9.2 | 13.8 | 0 | 84 | 26 |
| 1-Nov-20 | 28.2 | 29.4 | 9 | 13.3 | 0 | 88 | 24 |
| 2-Nov-20 | 29.2 | 29.2 | 11.6 | 13.3 | 0 | 81 | 29 |
| 3-Nov-20 | 28 | 28.7 | 11.5 | 12.8 | 0 | 74 | 38 |
| 4-Nov-20 | 28 | 28.6 | 10.6 | 13.1 | 0 | 89 | 36 |
| 5-Nov-20 | 29 | 28.3 | 10.5 | 12.5 | 0 | 78 | 39 |
| 6-Nov-20 | 28.5 | 28.5 | 9.5 | 12.3 | 0 | 93 | 51 |
| 7-Nov-20 | 27.4 | 28.1 | 9 | 12.2 | 0 | 88 | 30 |
| 8-Nov-20 | 28.2 | 27.8 | 9.5 | 12 | 0 | 90 | 45 |
| 9-Nov-20 | 27.2 | 27.9 | 9.2 | 11.8 | 0 | 93 | 39 |
| 10-Nov-20 | 27.4 | 27.7 | 10.6 | 11.6 | 0 | 86 | 36 |
| 11-Nov-20 | 27.6 | 27.5 | 9 | 11.4 | 0 | 91 | 38 |
| 12-Nov-20 | 26 | 27.2 | 8.4 | 11 | 0 | 93 | 40 |
| 13-Nov-20 | 27.6 | 27.2 | 8 | 11.1 | 0 | 91 | 32 |
| 14-Nov-20 | 22.6 | 27.3 | 11.4 | 11 | 0 | 93 | 86 |
| 15-Nov-20 | 19 | 26.9 | 12.5 | 10.7 | 0 | 87 | 94 |
| 16-Nov-20 | 20.5 | 26.5 | 12.4 | 10.2 | 27.8 | 95 | 54 |
| 17-Nov-20 | 23.8 | 26.1 | 8 | 9.9 | 0 | 95 | 49 |
| 18-Nov-20 | 23 | 26 | 8.2 | 10.4 | 0 | 95 | 51 |
| 19-Nov-20 | 21.6 | 26.4 | 8.4 | 10.1 | 0 | 95 | 38 |
| 20-Nov-20 | 22.6 | 26 | 6.8 | 9.6 | 0 | 94 | 43 |
| 21-Nov-20 | 21.2 | 25.8 | 4.6 | 9.5 | 0 | 94 | 49 |
| 22-Nov-20 | 19 | 25.5 | 4.5 | 9.4 | 0 | 97 | 90 |
| 23-Nov-20 | 20.6 | 25.2 | 9.4 | 9.2 | 0 | 95 | 54 |
| 24-Nov-20 | 19.6 | 25.1 | 13 | 8.9 | 0 | 82 | 55 |
| 25-Nov-20 | 17.2 | 24.8 | 12.8 | 8.8 | 0 | 86 | 62 |
| 26-Nov-20 | 22.6 | 24.8 | 8 | 8.1 | 8 | 97 | 45 |
| 27-Nov-20 | 25 | 24.3 | 6.2 | 8 | 0 | 87 | 37 |
| 28-Nov-20 | 23.4 | 24.1 | 5.6 | 8 | 0 | 95 | 45 |
| 29-Nov-20 | 22.8 | 24 | 9.4 | 7.7 | 0 | 92 | 68 |
| 30-Nov-20 | 23.8 | 23.9 | 7.6 | 7.7 | 0 | 90 | 48 |
| 1-Dec-20 | 24.2 | 24.1 | 7.8 | 7.8 | 0 | 94 | 47 |
| 2-Dec-20 | 24 | 23.7 | 7.2 | 7.7 | 0 | 92 | 48 |
